# The restoration ability of a short nap after sleep deprivation on the brain cognitive function: A dynamic functional connectivity analysis

**DOI:** 10.1111/cns.14413

**Published:** 2023-08-22

**Authors:** Ziliang Xu, Yingjuan Chang, Fan Guo, Chen Wang, Na Chai, Minwen Zheng, Peng Fang, Yuanqiang Zhu

**Affiliations:** ^1^ Department of Radiology, Xijing Hospital Fourth Military Medical University Xi'an China; ^2^ Department of Military Medical Psychology Fourth Military Medical University Xi'an China

**Keywords:** dynamic functional connectivity, psychomotor vigilance task, resting state functional magnetic resonance imaging, short nap, sleep deprivation

## Abstract

**Aims:**

The brain function impairment induced by sleep deprivation (SD) is temporary and can be fully reversed with sufficient sleep. However, in many cases, long‐duration recovery sleep is not feasible. Thus, this study aimed to investigate whether a short nap after SD is sufficient to restore brain function.

**Methods:**

The data of 38 subjects, including resting state functional magnetic resonance imaging data collected at three timepoints (before SD, after 30 h of SD, and after a short nap following SD) and psychomotor vigilance task (PVT) data, were collected. Dynamic functional connectivity (DFC) analysis was used to evaluate changes in brain states among three timepoints, and four DFC states were distinguished across the three timepoints.

**Results:**

Before SD, state 2 (a resting‐like FC matrix) was dominant (48.26%). However, after 30 h SD, the proportion of state 2 dramatically decreased, and state 3 (still resting‐like, but FCs were weakened) became dominant (40.92%). The increased proportion of state 3 positively correlated with a larger PVT “lapse” time. After a nap, the proportions of states 2 and 3 significantly increased and decreased, respectively, and the change in proportion of state 2 negatively correlated with the change in PVT “lapse” time.

**Conclusions:**

Taken together, the results indicated that, after a nap, the cognitive function impairment caused by SD may be reversed to some extent. Additionally, DFC differed among timepoints, which was also associated with the extent of cognitive function impairment after SD (state 3) and the extent of recovery therefrom after a nap (state 2).

## Graphical abstract

Sleep deprivation (SD) will lead to temporary cognitive impairment, which can be reversed to some extent by a short nap following SD. Through dynamic functional connectivity analysis, the dominant stationary brain states that the brain trends to be in differ before and after SD. The relative proportions of these two stationary brain states affected the degree of cognitive impairment after SD and recovery after a nap, respectively.

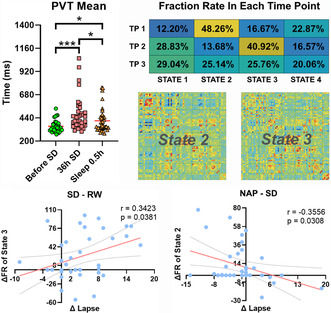

## 1 INTRODUCTION

Sleep deprivation (SD) is becoming increasingly common in our modern “24/7” society.^1^ SD has various consequences, including sleepiness, impaired cognitive performance, and even industrial, transportation, and medical accidents.^2^ Previous functional magnetic resonance imaging (fMRI) studies have documented the effects of SD on task‐related cerebral activation; in particular, changes in activity in prefrontal and parietal regions were induced by SD.^3^

The human brain is a complex network involving functional connectivity (FC) among various regions. Previous studies have indicated that SD upregulates connectivity in the default mode network (DMN) and impairs the functional network (FN), that is, the frontal–parietal network. Moreover, FC between the thalamus and cortical regions was compromised after SD.^4^ Our previous study using voxel‐mirrored homotopic connectivity (VMHC) revealed increased connectivity between the two brain hemispheres.^5^ Moreover, our recent study involving repeated fMRI scanning validated these findings and indicated that there is an interaction effect of circadian rhythmicity and homeostatic pressure on FC.^6^

The aforementioned findings were based on static FC. Dynamic functional connectivity (DFC) can reveal changes in patterns of brain connectivity that reliably occur across time and subjects.^7^, ^8^ To date, DFC has been used to investigate dynamic brain changes in the context of cognitive behavior and diseases,^9^, ^10^ but few studies have applied this method to investigate dynamic changes in FN in the context of SD. Li et al.^11^ investigated such changes during SD using DFC and showed that SD promoted a globally hypo‐connected state reflecting self‐focused processing, which would likely impair cognitive performance. Teng et al.^12^ used DFC and clustering analysis on the task‐free fMRI data and revealed five centroids, of which two were associated with high and low arousal, respectively.

The brain function impairment caused by SD is temporary and can be fully reversed with sufficient sleep. However, in many cases, long‐duration recovery sleep is not feasible, and rapid recovery from SD may be needed. In one study, a 90‐min afternoon nap helped restore hippocampal function.^13^ Furthermore, slow oscillatory transcranial direct current stimulation during a daytime nap improved mild cognitive impairment and helped patients consolidate memories.^14^ Thus, we posited that a short nap may be sufficient for the rapid reversal of SD‐induced brain function deficits.

In this study, DFC analysis was used to evaluate brain network changes among three timepoints (baseline, after 30 h of SD, and after a short nap following SD), and a FC matrix was derived through resting‐state fMRI (rs‐fMRI). At each timepoint, a psychomotor vigilance task (PVT) was performed after the rs‐fMRI scan. The PVT is a highly reproducible assay for vigilance that has been validated in multiple settings.^15^ In a previous study using the PVT to analyze changes in brain state among five timepoints during one night of total SD, we identified a timepoint at which changes in behavioral and imaging parameters reached their maximum during SD; thereafter, the differences gradually decreased, possibly due to circadian rhythmicity.^16^ These results demonstrated the sensitivity of the PVT to changes in brain state. In this study, DFC and PVT metrics were compared and correlated among the three timepoints to ascertain the degree of recovery. We hypothesized that (1) brain function can be partially recovered after a nap; (2) the baseline and SD timepoints would be associated with clearly dissociable brain states; and (3) after a nap, the brain state would show a marked change.

## 2 MATERIALS AND METHODS

### 2.1 Participants

Forty‐five right‐handed healthy subjects with nap habits were recruited for our study. Participants were excluded if they met any of the following criteria: (1) a history of psychiatric or neurologic disease; (2) the presence of a sleep disorder; (3) extreme morning or evening type, as assessed by a questionnaire^17^; (4) a job that required shiftwork; or (5) a history of alcohol or drug abuse. Subjects were required to record the number of hours they slept every night (during the week before the experiment) in a sleep diary. Only subjects with good sleep habits (>6.5 h of sleep per night, fell asleep no later than 1:00 a.m., and woke up no later than 9:00 a.m.) were invited to take part in the study.

All subjects provided informed written consent and the study was approved by the Ethics Committee of Xijing Hospital.

### 2.2 Study protocol

All participants made three visits to the laboratory. During the first visit, they were briefed about the experimental protocol and provided with a wristwatch (Mini‐Mitter Actiwatch; Philips Respironics) to record their sleep patterns.^18^ All participants signed the informed consent form. At the second visit, participants experienced 30 h of SD followed by a 30 min‐opportunity short nap. An MRI scan was done after SD and after a short nap. At the third visit, participants underwent an MRI scan after normal sleep (resting wakefulness [RW] or before SD). The order of the second and third visits was pseudo‐randomized to minimize the influence of the scanning sequence. To avoid a persistent effect of SD, the interval between the last two visits was at least 1 week. The SD process began at 8:00 a.m. and ended at 14:00 p.m. on the following day. During SD, participants were allowed to read, watch TV, or surf the internet, but the time was restricted to keep the participant from over‐vigilance (10:00–14:00 p.m. next day). Strenuous activities and caffeinated beverages were not allowed during the experiment. The temperature was around 23°C, and standard light conditions (340 lux) were used. Two researchers accompanied the subjects during SD and scanning to prevent them from falling asleep. For the RW condition, MRI scans were scheduled for 8:00–9:00 a.m., while for the SD condition, they were scheduled for 14:00–15:00 p.m. After completing the MRI scans in the SD condition, the participants were allowed to sleep for 30 min, after which they were woken up for the final MRI scan. Before that scan, a 20‐min break was provided for participants to refresh themselves to minimize the possible effects of sleep inertia. The duration of the nap was recorded using a wristwatch. According to the wristwatch, all participants slept for at least 20 min during the nap. After the nap, we also asked each participant if they were sleeping, and all of them answered that they fell asleep.

### 2.3 Psychomotor vigilance task

The ability to sustain attention was measured using the well‐defined PVT, which has been described in detail elsewhere.^15^ Briefly, at random intervals, a millisecond counter began to scroll, and participants were asked to press the space bar on a keyboard to stop the scrolling counter as quickly as possible. After pressing the button, the counter displayed the achieved RT for 1 s as feedback for monitoring their performances. The duration of the task was 10 min, and the inter‐stimulus intervals were distributed randomly from 2 to 10 s. The average stimuli trials (RW mean: 61.1 ± 1.38; SD mean: 60.2 ± 1.97) and the inter‐stimulus intervals (RW mean: 6.13 ± 0.61 s; SD mean: 6.09 ± 0.56 s) showed no significant differences between the two conditions.

### 2.4 MRI acquisition

All subjects underwent a series of scans performed with a 3T scanner (Discovery MR750; GE Healthcare) at the Department of Radiology, Xijing Hospital, Fourth Military Medical University, Xi'an, China. A standard 8‐channel head coil was used together with a restraining foam pad to minimize head motion and scanner noise. Resting‐state functional images were acquired at each timepoint using a single‐shot gradient‐recalled echo planar imaging sequence. For each subject, a total of 210 volumes were acquired (repetition time [TR]/echo time [TE]: 2000 ms/30 ms, field of view: 240 × 240 mm^2^, matrix size: 64 × 64, flip angle: 90°, in‐plane resolution: 3.75 × 3.75 mm^2^, slice thickness: 3.5 mm with no gaps, 45 axial slices). High‐resolution T1‐weighted images were collected using a volumetric three‐dimensional spoiled gradient recall sequence (TR/TE: 8.2 ms/3.18 ms, field of view: 256 × 256 mm^2^, matrix size: 512 × 512, flip angle = 9°, in‐plane resolution: 0.5 × 0.5 mm^2^, slice thickness = 1 mm, 196 sagittal slices) at the first timepoint, that is, before SD. During the resting scan, the subjects were instructed to keep their eyes open and not think about anything.

### 2.5 Data processing

**FIGURE 1:**
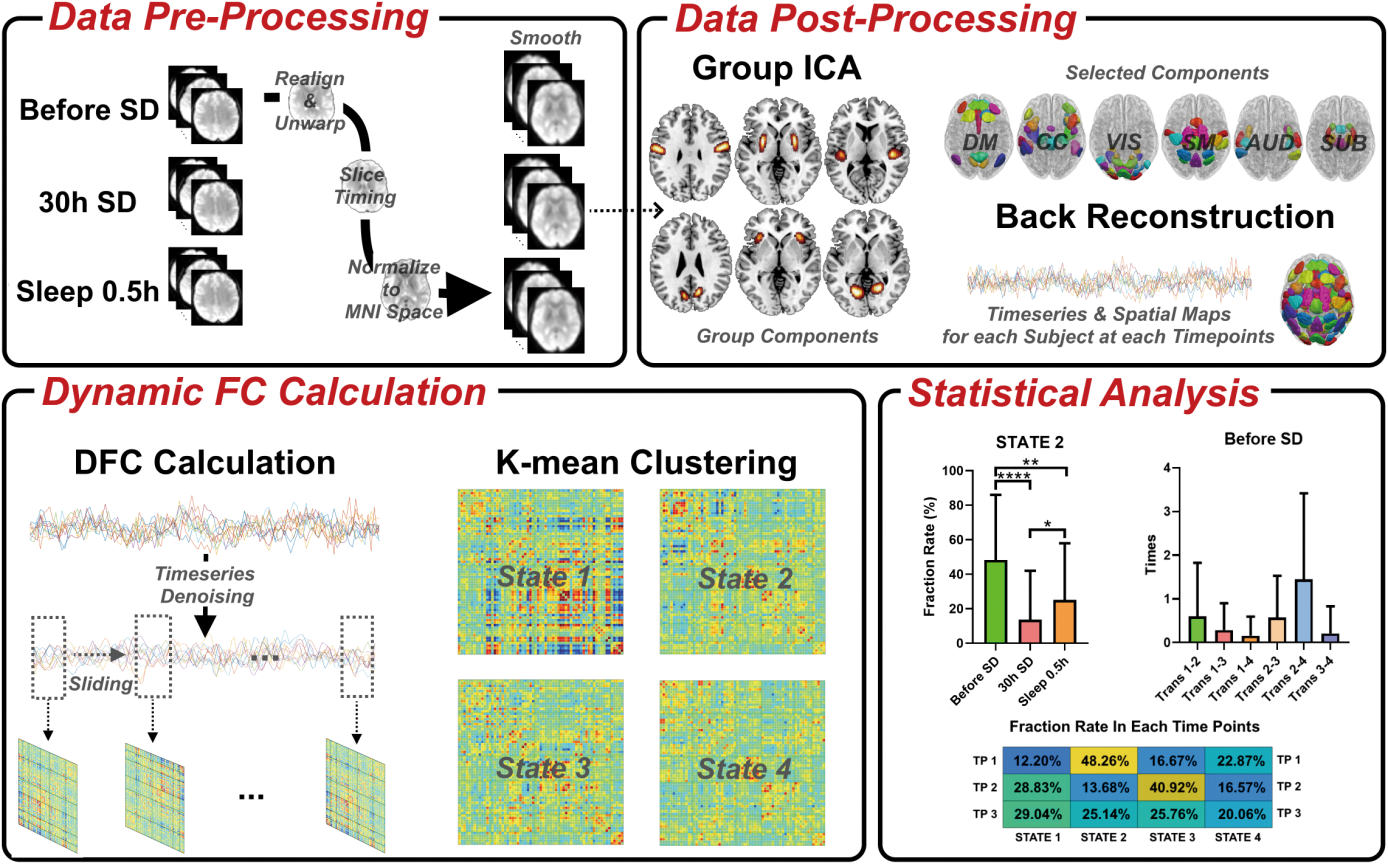
Workflow of the dynamic functional connectivity calculation procedure, including data pre‐processing, data post‐processing, dynamic FC calculation, and statistical analysis. FC, functional connectivity.

Data processing steps includes (1) data pre‐processing, (2) group independent component analysis (GICA), (3) DFC calculation and (4) DFC state clustering. Detailed information is described in Appendix S1. The detailed workflow of this study is displayed in Figure 1.

Magnetic resonance imaging data were pre‐processed using the CONN FC toolbox (https://www.nitrc.org/projects/conn/). GICA, DFC calculation, and state clustering were performed on pre‐processed fMRI images using GIFT software (https://trendscenter.org/software/gift/).

### 2.6 Evaluation metrics

In this study, the fraction rate, dwell time, and transition time^19^ were calculated to compare DFC states among three timepoints. The fraction rate is the occurrence ratio (proportion) of each DFC state, that is, the ratio between the frequency of each DFC state and the total number of DFC states. The dwell time is the number of consecutive occurrences of each DFC state. The transition time is the number of transitions between each pair of DFC states. The fraction rate is useful for visualizing the relative proportions of each DFC state at each timepoint, while the dwell time assesses the persistence of individual DFC states and the transition time evaluates the stability of each state.

Three additional metrics derived from graph theory, that is, global efficiency, nodal eccentricity, and local efficiency, were also used to evaluate the DFC states. Global efficiency refers to the overall network efficiency (i.e., interaction ability) of each state. Nodal eccentricity and local efficiency pertain to the efficiency of nodes within the network in each state.

Psychomotor vigilance task metrics including mean, median, maximum, minimum response, and lapse times (reactions longer than 500 ms) were recorded at each timepoint to evaluate the cognitive state of the participants.

### 2.7 Statistical analysis

All statistical analyses were performed using MATLAB software (MathWorks Inc.) with in‐house scripts. Summary statistics are presented as counts (percentages) for categorical variables, as mean ± standard deviation for normally distributed continuous variables, and as middle (quartiles) for non‐normally distributed continuous variables. Data distribution was checked using the Shapiro–Wilk test. The chi‐square test or Fisher exact test was used to compare categorical variables. A repeated‐measures one‐way analysis of variance (ANOVA) or non‐parametric repeated‐measures one‐way ANOVA was used to compare the fraction rate, transition time metrics, and PVT metrics among the three timepoints. One‐way ANOVA or non‐parametric one‐way ANOVA was used to compare dwell time, global efficiency, nodal eccentricity, and local efficiency metrics among the three timepoints, where none of these four metrics could take a value of zero if a subject did not have data for one or more DFC states. For each metric, a post‐hoc *t*‐test or non‐parametric test (Bonferroni‐corrected) was performed if the ANOVA result was statistically significant. Pearson correlation adjusted for age, gender, and body mass index (BMI) was used to assess the relationships between DFC and PVT metrics. The threshold for statistical significance was set at *p* < 0.05.

## 3 RESULTS

### 3.1 Demographic characteristics and PVT information

The detailed demographic information and PVT task information are illustrated in Table 1. After data preprocessing, 38 subjects remained for further analysis.

**TABLE 1:** Demographic characteristics and PVT performances.

Gender (male/female)	20/18
Age (years)	20.71 ± 2.12
Body mass index	21.90 ± 0.64
Duration of nap (min)	25.74 ± 2.60
PVT performance	
Before sleep deprivation	
Mean reaction time (ms)	352.72 ± 60.29
Middle reaction time (ms)	335.93 ± 49.70
Maximum reaction time (ms)	472.86 ± 140.49
Minimum reaction time (ms)	280.26 ± 29.88
Lapse times	3.68 ± 6.04
After sleep deprivation	
Mean reaction time (ms)	472.45 ± 175.64
Middle reaction time (ms)	367.92 ± 43.82
Maximum reaction time (ms)	947.86 ± 767.25
Minimum reaction time (ms)	299.04 ± 32.16
Lapse times	7.84 ± 6.55
After a nap	
Mean reaction time (ms)	409.00 ± 125.93
Middle reaction time (ms)	357.30 ± 55.78
Maximum reaction time (ms)	680.54 ± 485.23
Minimum reaction time (ms)	288.64 ± 30.93
Lapse times	6.39 ± 8.37

Abbreviation: PVT, psychomotor vigilance task.

### 3.2 Brain network mapping

After group‐level ICA, we extracted 65 ICs from 100 components and used them to construct six brain FC networks (Figure S1): a default mode (DM) network (13 ICs), a cognitive control (CC) network (17 ICs), a visual (VIS) network (10 ICs), a somatomotor (SM) network (13 ICs), an auditory (AUD) network (7 ICs), and a sub‐cortical (SC) network (5 ICs). The DM network consisted of the superior and middle frontal gyri (SFG and MFG, respectively), anterior cingulate cortex (ACC), angular gyrus (AG), and precuneus. The CC network consisted of the inferior frontal gyrus (IFG), MGF, inferior parietal gyrus (IPG), middle and inferior temporal gyrus (MTG and ITG, respectively), supramarginal, insula, and hippocampus. The VIS network consisted of the MTG, ITG, superior, middle, and inferior occipital gyri (SOG, MOG, and IOG, respectively), lingual gyrus (LG), fusiform, calcarine, and cuneus. The SM network consisted of the pre‐ and post‐central gyrus (PreCG and PostCG, respectively), supplementary motor area (SMA), paracentral lobule (PL), and superior parietal gyrus (SPG). The AUD network consisted of the superior temporal gyrus (STG), MTG, and superior temporal pole (STP). The SC network consisted of the putamen, pallidum, caudate, and thalamus. The static FC map is shown in the right part of Figure S1.

### 3.3 Dynamic functional connectivity states

**FIGURE 2:**
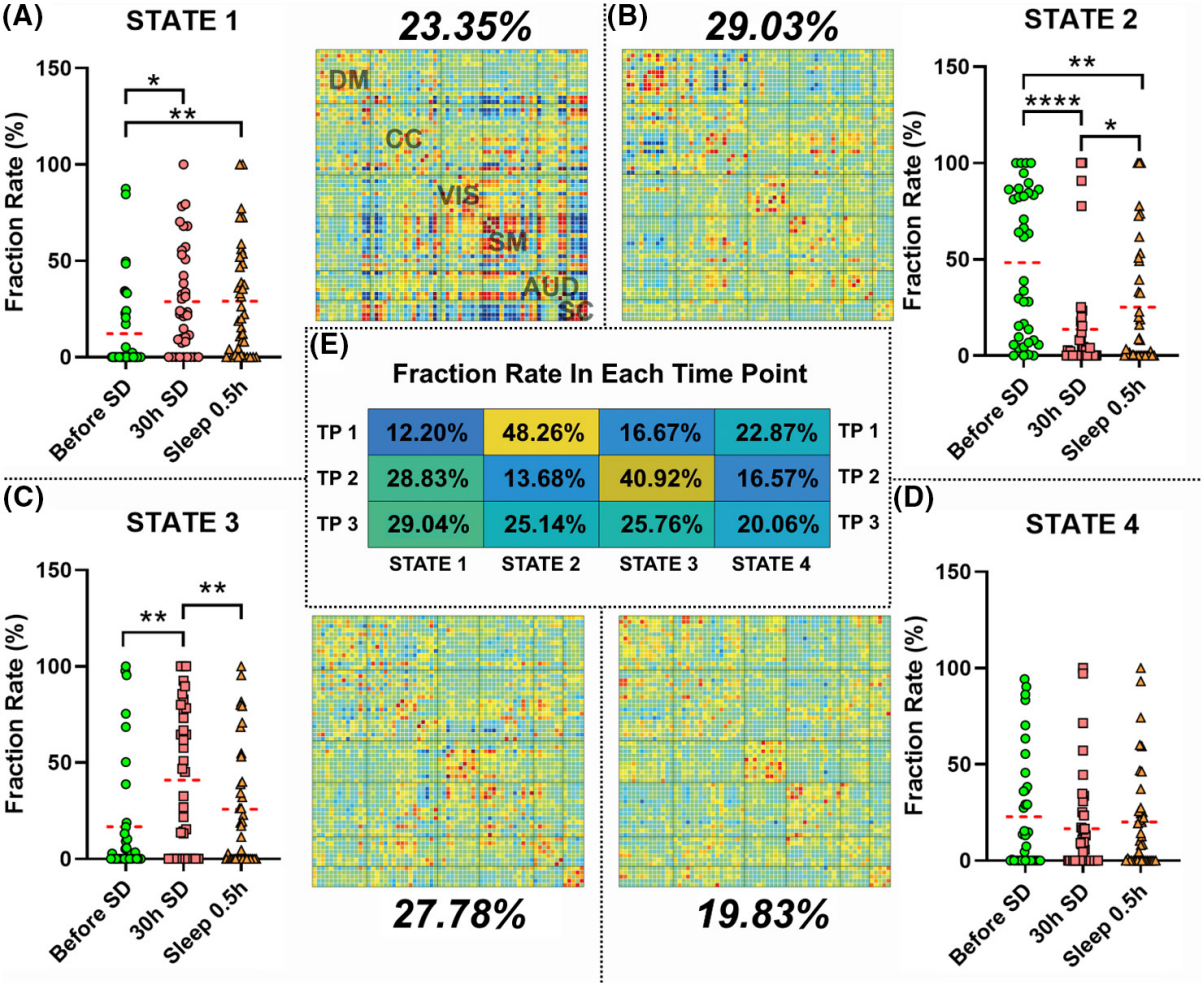
Fraction rate comparison among timepoints for state 1–4 (A–D), and the four dynamic functional connectivity states (E). *p < 0.05, **p < 0.01, ****p < 0.0001.

K‐means clustering yielded four DFC state clusters based on the elbow criterion (Figure 2). State 2 was dominant (29.03%), followed by state 3 (27.38%), state 3 (23.35%), and state 4 (19.83%). State 2 equated to “normal”, that is, resting state FC, with a negative FC value seen between the DM and CC networks, and a positive FC value seen within each network. State 1 exhibited relatively stronger FC within and between networks than state 2.

### 3.4 Dynamic functional connectivity metrics

As shown in Figure 2, before SD, states 2–4 were the dominant DFC states, with states 2 and 1 accounting for 48.26% and 12.20% of the total DFC, respectively. After 30 h of SD, the proportions of states 1 and 3 had significantly increased; state 3 reached its highest proportion (40.92%). After a short nap, the proportion of state 2 had significantly increased, and that of state 3 had significantly decreased.

**FIGURE 3:**
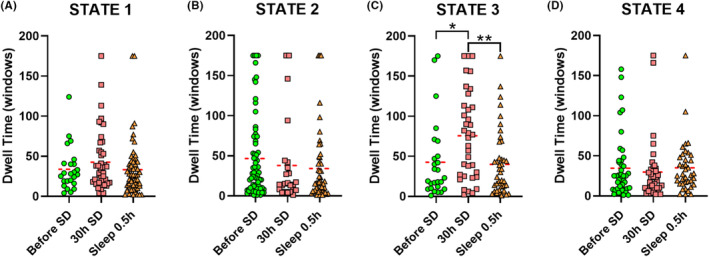
Dwell time among timepoints for state 1–4 (A–D). *p < 0.05, **p < 0.01.

**FIGURE 4:**
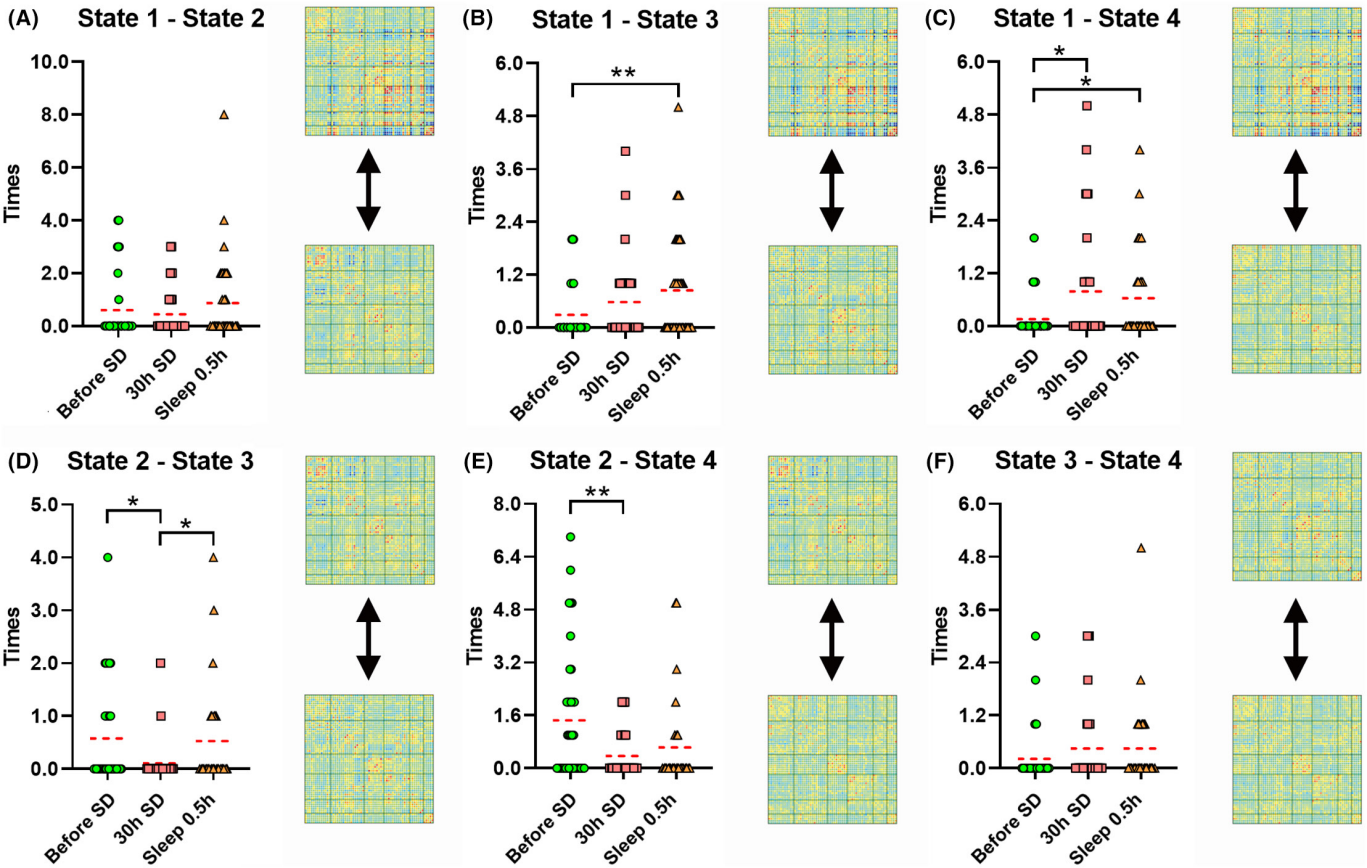
Transition time among timepoints for (A) state 1–2, (B) 1–3, (C) 1–4, (D) 2–3, (E) 2–4 and (F) 3–4. *p < 0.05, **p < 0.01.

As shown in Figure 3, the dwell time of state 3 after 30 h of SD was significantly longer than for the other two timepoints. As shown in Figure 4, the transition time between states 1 and 4 before SD was significantly lower than for the other two timepoints. The transition time between states 1 and 3 before SD was significantly lower than that after a short nap. The transition time between states 2 and 3 after 30 h of SD was significantly lower than that between the other two timepoints. Finally, the transition time between states 2 and 4 before SD was significantly larger than that after 30 h of SD.

Regarding the graph theory metrics, the global efficiency, local efficiency, and nodal eccentricity of states 2–4 exhibited no statistical difference among the three timepoints (Figure S2). However, for state 1, the global and local efficiency after 30 h were significantly smaller than at the other timepoints, while the nodal eccentricity was significantly larger.

### 3.5 PVT task

**FIGURE 5:**
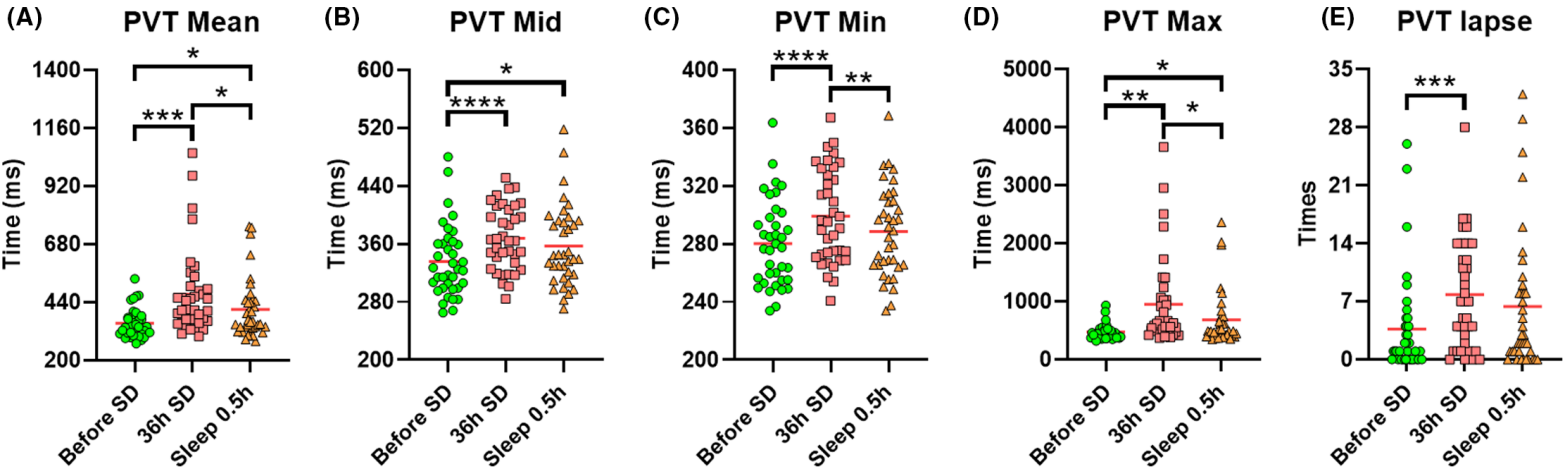
(A) Mean, (B) median, (C) minimum, (D) maximum, and (E) lapse time of PVT task among timepoints. *p < 0.05, **p < 0.01, ***p < 0.001, ****p < 0.0001.

Figure 5 shows that, after SD, all PVT metrics significantly increased. After a nap, the mean, minimum, and maximum PVT response times significantly decreased, which reflects a partial recovery of cognitive and attentional function.

**FIGURE 6:**
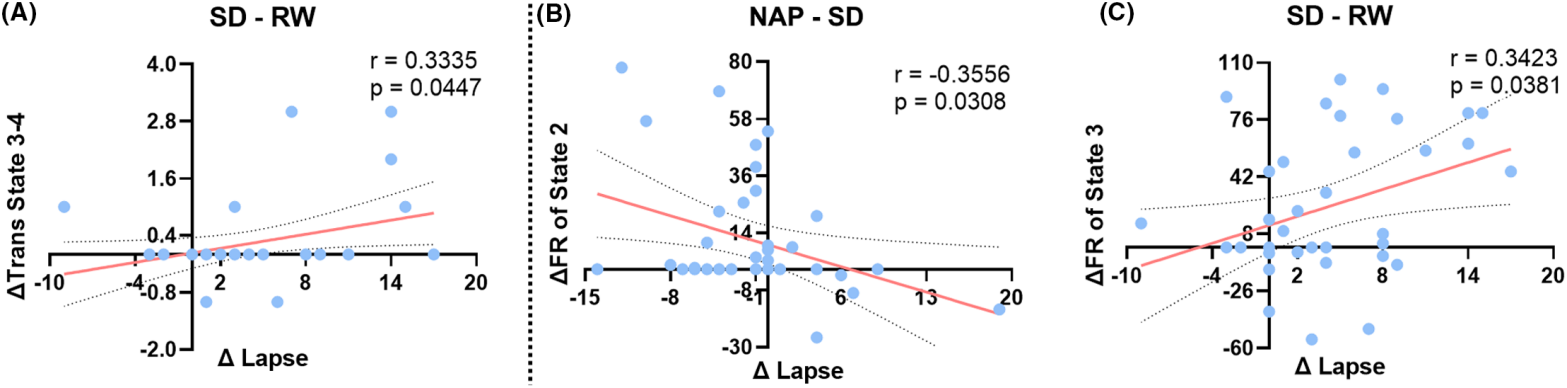
Correlations between dynamic functional connectivity and psychomotor vigilance task metrics after adjusted for age, gender, and BMI. (A) Between Δ Trans state 3‐4 and Δ lapse (SD‐RW), (B) between Δ FR of state 2 and Δ lapse (NAP‐SD) and (C) between Δ FR of state 3 and Δ lapse (SD‐RW). “Δ Trans State” indicates the transition time between states. FR, fraction rate; RW, resting state wakefulness, that is, before SD; SD, sleep deprivation.

Figure 6 illustrates the correlations between DFC and PVT metrics. After being adjusted for age, gender, and BMI, the change in transition time between states 3 and 4 positively correlated with the change in PVT lapse time (SD‐RW). The change in proportion of state 3 positively correlated with the change in PVT lapse time after SD (SD‐RW), while the change in proportion of state 2 negatively correlated with the change in PVT response time after a nap (Nap‐SD).

## 4 DISCUSSION

In this study, brain recovery after a short nap following SD was investigated. Dynamic changes in brain state among three timepoints (before SD, 30 h after SD, and after a short nap following SD) were captured by DFC analysis in terms of the fraction rate, dwell time, transition time, and three graph theory metrics. Differences in dominant DFC states were observed among timepoints before and after SD (states 2 and 3, respectively). Furthermore, the changes in DFC metrics after SD indicated cognitive function impairment, and this could be reversed to some extent after a nap (reflected in improvements in PVT performance). A detailed discussion of these results follows.

Dynamic functional connectivity is very useful for evaluating changes in brain state. Before SD, state 2 accounted for about half of all DFC, while state 1 accounted for the smallest proportion among states. In state 1, abnormally strong connective strength within the SM and SC networks and between the SC network and other networks was found. Considering its relatively low proportion, state 1 may be regarded as a “non‐stationary state”. In state 2, a relatively strong positive connection was found within each network and a strong negative connection between the DM, SC, and SM networks, which is more “resting‐like”. Thus, state 2 may be regarded as a “stationary state” that predominates before SD. After 30 h of SD, state 3 was the dominant state, and the state 3 to state 2 ratio was approximately equal to the state 2 to state 3 ratio before SD. Compared with state 2, all connection strengths within and between each network were weakened in state 3, but still somewhat “resting‐like”. Thus, state 3 may be regarded as the stationary state after SD, which also indicates that the timepoints before and after SD may differ in terms of the stationary state and may indicate the temporal impairment of cognitive and other normal brain functions. These results were very similar to those of a study showing that stationary states differed between patients and healthy controls^20^ and suggest that 30 h of SD alters the “stationary state” of subjects to some extent (as shown in other resting state studies of mental disorders^21^, ^22^). As state 4 had the weakest connection strength within and between each network among all states in this study, it may represent a transitory state between the “stationary” and “non‐stationary” states.

Sleep deprivation can lead to temporary cognitive impairment. In one study, 24 h of SD led to impairments in cognitive performance, reflected in longer reaction times and less capacity for sustained attention.^23^ Also, our previous study provided evidence that different cognitive tasks are differentially affected by sleep loss.^6^ In this study, this phenomenon seemed to be reflected in brain state changes. Before SD, the most resting state‐like state (i.e., state 2) was dominant, while the proportion of the “non‐resting state‐like” state (i.e., state 1) was the smallest among all states. These results suggest that subjects can easily maintain wakefulness before SD. After 30 h of SD, however, the proportion of state 2 significantly decreased, and state 3 became dominant. Compared to state 2, the connectivity in the DM and between the DM and CC networks was relatively low in state 3 (i.e., trended toward zero, reflected in a green color for the state 3 matrix). Dysconnectivity in the DM and between the DM and CC networks, has been reported previously in Alzheimer's disease and attention deficit hyperactivity disorder patients.^24^, ^25^ Meanwhile, the connectivity of the CC network was also relatively low. The CC network is involved in task execution and behavioral adaptation,^7^ and the degree of connectivity within this network directly affects cognition function.^26^ Our results suggest that, after SD, sleepiness leads to dysconnectivity in the DM and CC and between the DM and CC networks, which in turn causes cognitive deficits. After SD, the proportion of the non‐resting state (state 1) was also significantly increased. Furthermore, switching between the non‐stationary (state 1) and stationary (state 3) states and between stationary states (states 2 and 3) showed a trend toward an increase and decrease, respectively. These results suggest that, with greater sleepiness, subjects had to fend off sleep more frequently, which may have affected their cognitive function. Additionally, all PVT performance metrics were significantly increased after SD, which indicates cognitive and attentional impairments. Interestingly, the change in proportion of state 3, and the transition between state 3 and transitory states 4 positively correlated with the change of PVT lapse time. These results suggest that state 3 is the stationary state after SD; however, the increasing dominance of state 3 after SD can exacerbate cognitive function impairment.

After a nap, although the proportion of state 1 showed no significant change compared with that after SD, the proportion of state 2 was significantly increased, while the proportion and dwell time of state 3 were significantly decreased. Switching between stationary states was also increased. These results may suggest that, after a nap, although subjects still needed to fend off sleep frequently (since the proportion of state 1 was not significantly decreased), the brain state was returning to baseline (although it was still some way off full recovery). Interestingly, only the global efficiency, local efficiency, and nodal eccentricity of state 1 differed significantly among the three timepoints. The global and local efficiency were significantly lower after SD, while the nodal eccentricity was significantly greater, suggesting lower network efficiency after SD. Network efficiency impacts task execution and performance.^27^ These results suggest that, after SD, sleepiness decreased network efficiency, and this was significantly ameliorated by a nap. Thus, although subjects still needed to fend off sleep frequently, executive cognitive function appeared to be improved compared with that after SD. The mean, maximum, and minimum PVT response times were also significantly decreased after a nap, which further supports the recovery of cognitive and attentional function. However, the mean and maximum response times were still significantly longer than before SD, thus reinforcing the notion of only partial recovery of cognitive function. Finally, the change in proportion in state 2 negatively correlated with the change in PVT lapse time. These results suggest that the larger the relative proportion of state 2 after a nap, the greater the degree of cognitive function recovery; thus, the proportion of state 2 may be key to the cognitive function recovery that occurs after a nap following SD.

Our study had some limitations. First, the number of subjects was relatively small. Second, the proportion of state 1 significantly increased after SD but not significantly decreased after a nap, which may indicate a recovery of cognitive function only to some extent. However, due to the very strange connections within and between networks in this state, future studies will focus on the function of this brain connection mode. Third, our finding that a nap after SD improved the brain state and cognitive function, as supported by changes in PVT performance metrics, still requires validation of other cognitive tasks. Finally, as the nap is a key component of the study, it is essential to investigate whether those who sleep less than 30 min will show less effects on state changes, whether the dream prior to wake‐up will affect the extent of cognitive function recovery, and the effect of different nap times on state changes, etc. Future studies will use polysomnography to objectively investigate the effects of different nap durations and nap structures (such as dream and special sleep waves) on cognitive function.

In summary, through DFC analysis, brain state changes among three timepoints (before SD, after 30 h of SD, and after a short nap following SD) were evaluated. The results showed that SD led to temporary cognitive impairment, which was reversed to some extent by a nap. Additionally, stationary states differed after (state 3) and before SD (state 2), and their proportional changes affected the degree of cognitive impairment after SD and recovery after a nap, respectively.

## FUNDING INFORMATION

This study was financially supported by the Key R&D Program Projects of Shaanxi, China (grant numbers 2022JM‐575 and 2021SF‐287); Boost Program of Xijing Hospital (grant numbers XJZT21CM21, JSYXZ08, and JSYXM28); Military Medical Science and Technology Youth Training Program (grant numbers 20QNPY049); National Natural Science Foundation of China (grant numbers 81801772, 81902488, and 82071917); and Youth Talent Lifting Project of Shaanxi province (F.G. grant number 20210304).

## CONFLICT OF INTEREST STATEMENT

The authors declare no conflicts of interest.

## Supporting information


Appendix S1

## Data Availability

The data that support the findings of this study are available on request from the corresponding author. The data are not publicly available due to privacy or ethical restrictions.
