# Supplementary material for: The restoration ability of a short nap after sleep deprivation on the brain cognitive function: A dynamic functional connectivity analysis
Source: CNS Neurosci Ther. 2023 Aug 22;30(2):e14413. doi: 10.1111/cns.14413 (PMC10848048; doi:10.1111/cns.14413)
Supplement: Supplementary file 1 — Appendix S1 [file CNS-30-e14413-s001.zip › supplementary file.docx]

## MATERIALS & METHODS

### Data Pre-processing

MRI data were pre-processed using the CONN FC toolbox (*https://www.nitrc.org/projects/conn/*). The Default Pre-processing Pipeline was used. Briefly, structural T1-weighted images were segmented into grey matter (GM), white matter (WM) and cerebral spinal fluid (CSF) images. Then, the images were normalized to Montreal Neurological Institute (MNI) space and used for the subsequent voxel-based denoising.

For fMRI, the first five volumes were excluded to ensure that the signal had reached equilibrium. The functional images were then corrected for head motion and temporal differences. We excluded the data of participants with translation or rotation exceeding ±1.5 mm and ±1.5°, respectively, at any timepoint; 38 subjects were included in the final analysis. Next, outliers were detected on corrected images. Then, functional images were normalized to MNI space, resampled at 2 × 2 × 2 mm^3^ and smoothed with a 6-mm full width at half maximum Gaussian kernel. Finally, the timeseries of each brain voxel was z-scored to control for variance, i.e., to minimize possible bias prior to data reduction [1].

### Group Independent Component Analysis

Group independent component analysis (GICA) was performed on pre-processed fMRI images using GIFT software (*https://trendscenter.org/software/gift/*). The processing steps were similar to a previous report [1]: 1) subject-specific principal component analysis (PCA) was performed on pre-processed fMRI images using a standard decomposition algorithm, with 120 principal components (PCs) retained for each subject at each timepoint; 2) group-level PCA was performed to reduce the 14,760 initial PCs (41 subjects × 3 timepoints × 120 PCs) to 100 using the expectation–maximization (EM) algorithm [2]; 3) GICA was performed on the 100 PCs to decompose them into 100 independent components (ICs) using ICASSO (*http://www.cis.hut.fi/projects/ica/icasso*), with the Infomax independent component analysis (ICA) algorithm [3] being repeated 20 times; and 4) spatial-temporal regression (back reconstruction) was performed on the 100 ICs, to transform them into 100 subject-specific ICs with corresponding timeseries for each subject at each timepoint. After the GICA, we manually checked the spatial maps for each IC and removed ICs related to WM, CSF, cerebellum or image artifacts. Finally, 65 ICs were selected for further analysis; six brain FC networks were constructed including a default mode (DM) network (13 ICs), cognitive control (CC) network (17 ICs), visual (VIS) network (10 ICs), somatomotor (SM) network (13 ICs), auditory (AUD) network (7 ICs) and sub-cortical (SC) network (5 ICs).

### Dynamic Functional Connectivity Calculation

For dynamic functional connectivity (DFC) calculation, GIFT software was used. The analysis process included three main steps: 1) timeseries denoising of each IC and 2) DFC calculation

For each subject at each timepoint, the timeseries for each IC was denoised via the following steps: 1) linear, quadratic, and cubic detrending; 2) multiple regression of six head motion parameters and averaging WM and CSF signals and outliers signal (exported from CONN software); and 3) 0.15 Hz lowpass filtering. After denoising, DFC was analyzed using a sliding window method. A rectangular window with a length of 30 TRs, Gaussian (σ) of 3 TRs, and sliding step of 1 TR was applied to the timeseries of 65 ICs. In each window, a 65 × 65 FC matrix was estimated from the regularized precision matrix [4,5] to prevent effects of the relatively short segments on the FC matrix. The L1 norm penalty was also placed on the precision matrix to further improve the sparsity of the FC matrix. For each subject at each timepoint, a 65 × 65 × 175 DFC matrix was calculated.

### Dynamic Functional Connectivity State Clustering

Finally, the k-means clustering algorithm [6] was applied to the DFC matrices to determine the frequency and structure of recurring FC states. Only FC matrices from timepoints for which the variance of all FC coefficients reached the local maxima along the time axis (subject exemplar) were selected as inputs [1]. A k-means clustering algorithm with L1 distance was repeated 500 times on subject exemplars to increase the likelihood of escaping the local minima, with random initialization of centroid positions at each timepoint. The resulting centroids (cluster medians) were then used for the initialization of centroid positions to cluster all DFC matrices (38 subjects × 3 timepoints × 175 windows = 19,950 instances). The optimal number of clusters was estimated as four based on the elbow criterion, which corresponds to four reoccurring FC states.

## REFERENCES

1. Allen EA, Damaraju E, Plis SM, Erhardt EB, Eichele T, Calhoun VD. Tracking whole-brain connectivity dynamics in the resting state. Cereb Cortex, 2014, 24(3): 663-76.
2. Roweis S. EM algorithms for PCA and SPCA. Neural Inform Process Syst, 1998, 10:626–632.
3. Bell AJ, Sejnowski TJ. An information-maximization approachto blind separation and blind deconvolution. Neural Comput, 1995, 7: 1129–1159.
4. Varoquaux G, Gramfort A, Poline JB, Thirion B. Brain covariance selection: better individual functional connectivity models using population prior. Advances in neural information processing systems, Vancouver, Canada: 2010.
5. Smith SM, Miller KL, Salimi-Khorshidi G, Webster M, Beckmann CF, Nichols TE et al. Network modelling methods for FMRI. Neuroimage, 2011, 54: 875–891.
6. Lloyd S. Least squares quantization in PCM. IEEE Trans Inform Theory, 1982, 28: 129–137.

## FIGURES


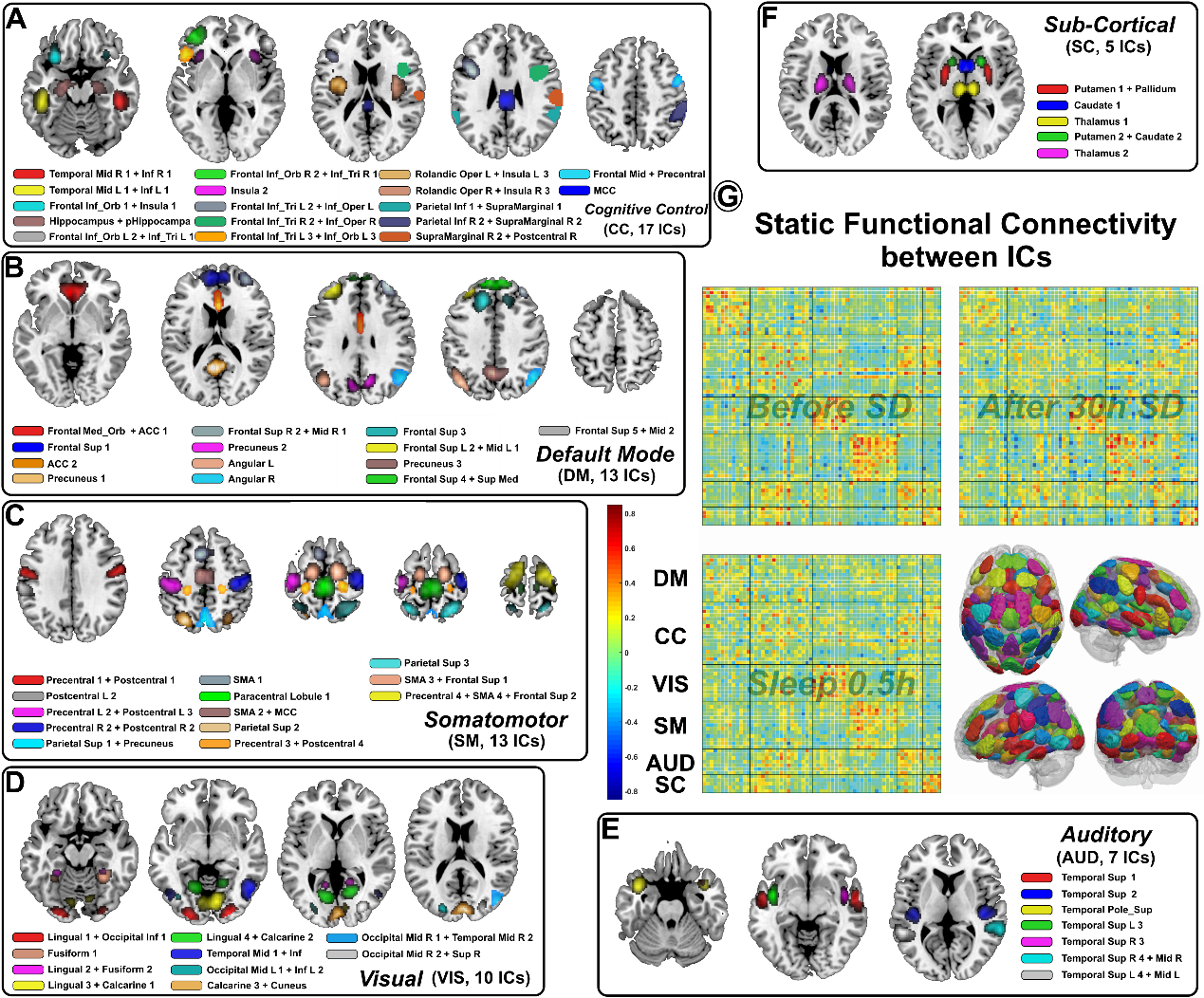


**Figure S1** Six brain networks (A for cognitive control, B for default mode, C for somatomotor, D for visual, E for auditory and F for sub-cortical networks) and (G) the functional connectivity map.


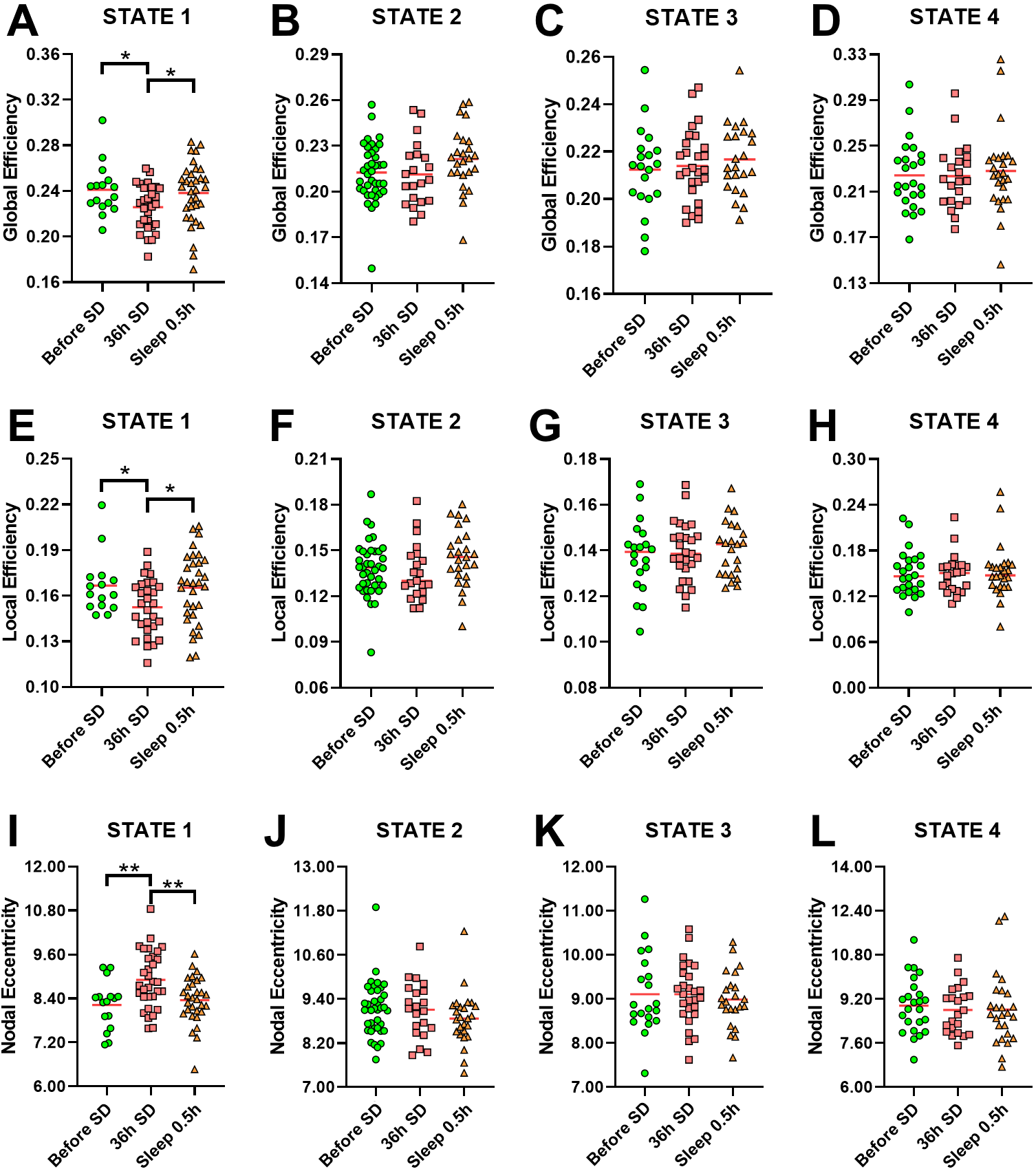


**Figure S2** Global efficiency (A-D), local efficiency (E-H) and nodal eccentricity (I-L) among timepoints for state 1 to state 4. * P < 0.05, ** P < 0.01.
