# Supplementary figures and images for: The restoration ability of a short nap after sleep deprivation on the brain cognitive function: A dynamic functional connectivity analysis
Source: CNS Neurosci Ther. 2023 Aug 22;30(2):e14413. doi: 10.1111/cns.14413 (PMC10848048; doi:10.1111/cns.14413)

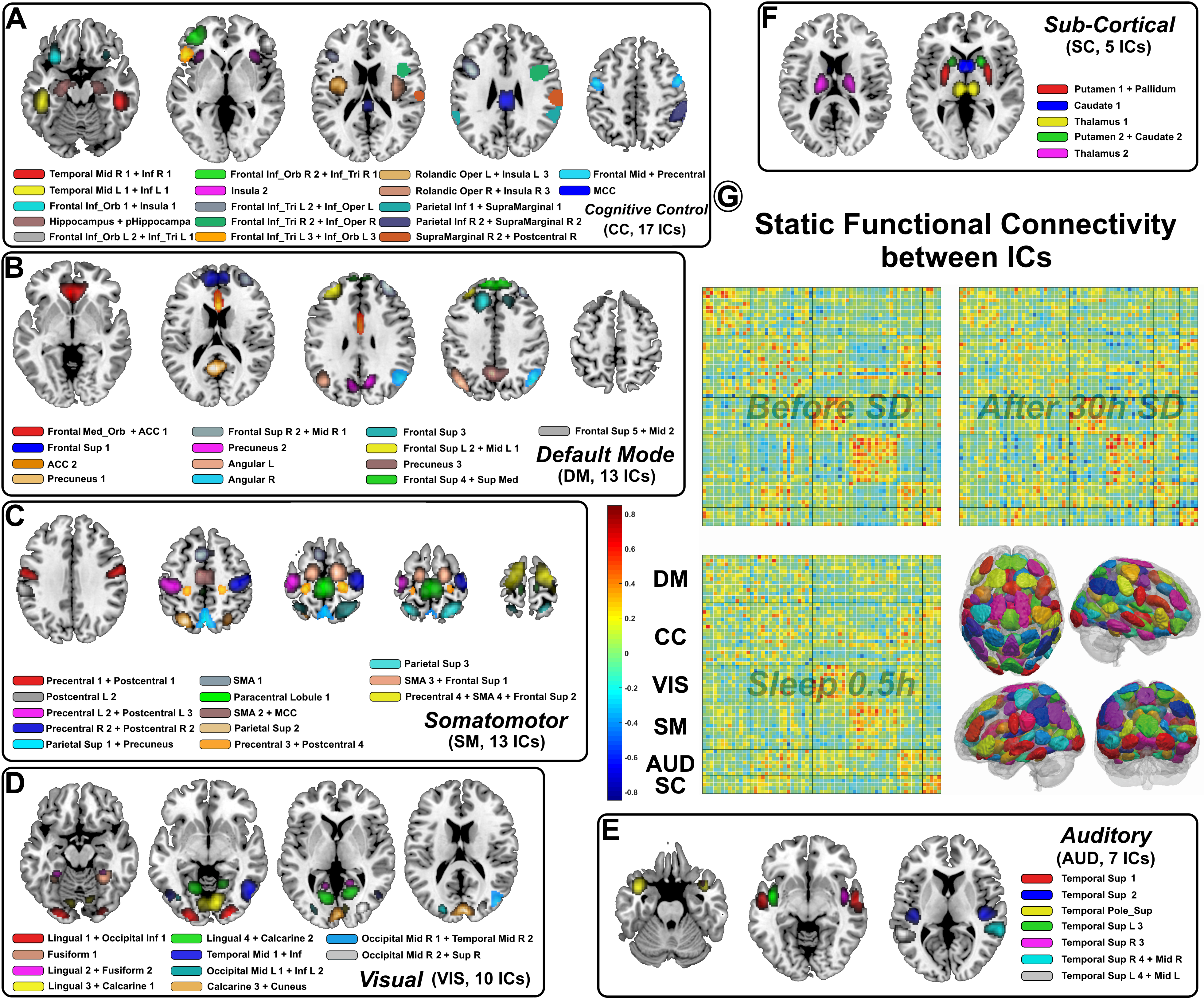

Supplement: Supplementary file 1 — Appendix S1 [file CNS-30-e14413-s001.zip › figure S1-50%-ID.tiff]

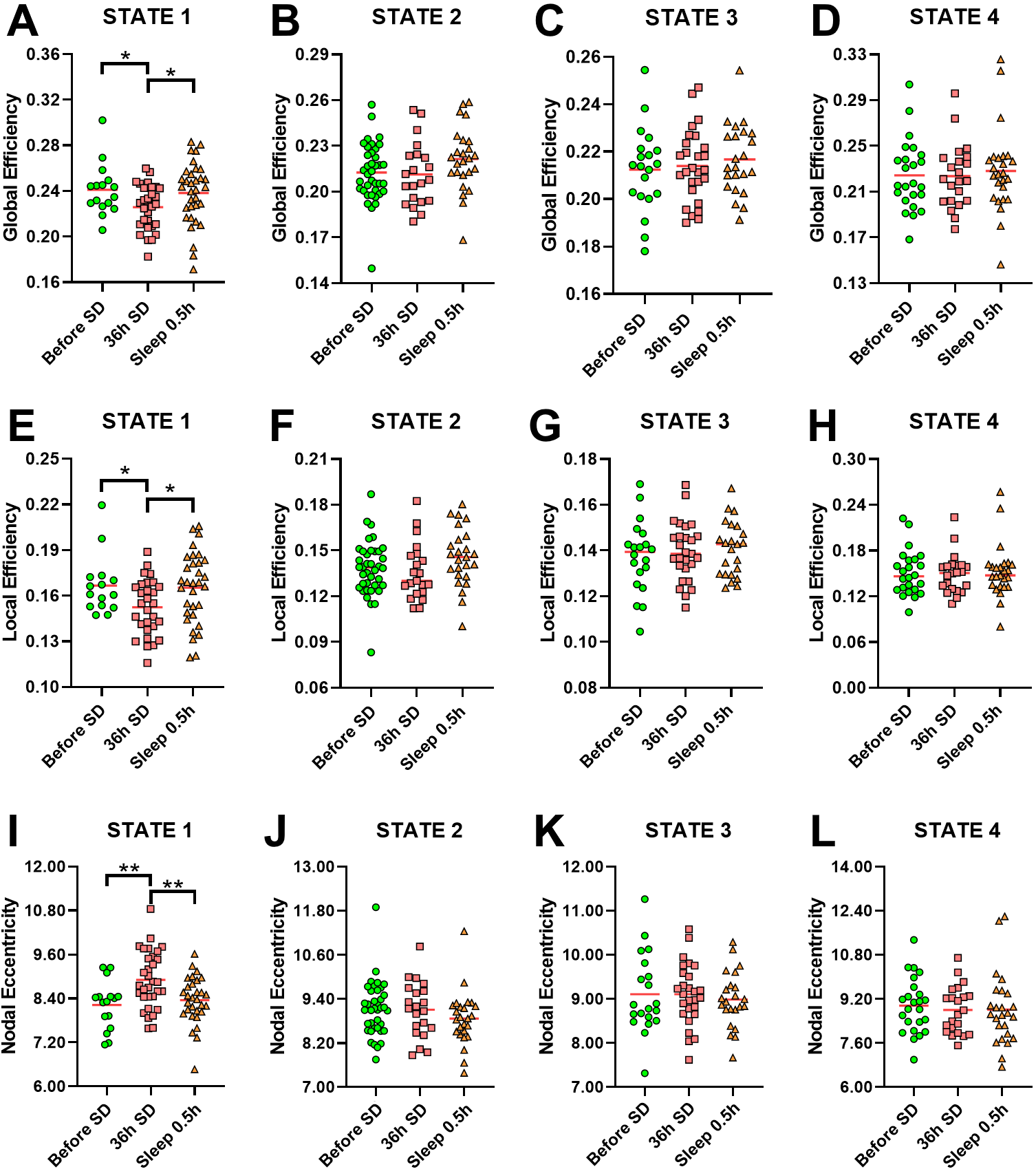

Supplement: Supplementary file 1 — Appendix S1 [file CNS-30-e14413-s001.zip › Figure S2-ID.tiff]
